# Barriers to Timely Lung Cancer Screening Among High-Risk Populations in Saudi Arabia: A Cross-Sectional Survey

**DOI:** 10.7759/cureus.83178

**Published:** 2025-04-29

**Authors:** Ghaida Alsuhim, Razan Alwabel, Thuraya Alshaikhi, Yaseer AlAnazi, Njood Alsudairy

**Affiliations:** 1 General Practice, King Khalid University, Abha, SAU; 2 General Practice, King Faisal University, Hofuf, SAU; 3 Radiology, Jeddah Second Health Cluster, Jeddah, SAU

**Keywords:** access, awareness, barriers, high-risk populations, low-dose computed tomography, lung cancer, saudi arabia, screening, stigma

## Abstract

Background

Lung cancer is a leading cause of cancer-related mortality worldwide, primarily driven by smoking. Although low-dose computed tomography screening effectively reduces lung cancer mortality through early detection, participation in screening programs remains low, particularly among high-risk populations. In Saudi Arabia, data on barriers to screening uptake are limited. This study aimed to identify and evaluate the barriers to timely lung cancer screening among high-risk individuals in Saudi Arabia, including awareness levels, access issues, personal attitudes, and perceived obstacles.

Methodology

A cross-sectional survey was administered between January and March 2025 to high-risk individuals in Saudi Arabia, defined by either a smoking history or a family history of lung cancer. Participants were recruited from multiple healthcare centers using convenience sampling. The survey assessed demographic information, knowledge and awareness of screening, perceived barriers, access to services, and attitudes toward screening.

Results

Of the 200 participants, 112 (56.0%) were male and 88 (44.0%) were female. Full awareness of lung cancer screening programs was reported by 72 (36.0%) participants, while 63 (31.5%) were unaware. Key reported barriers included lack of awareness (28.0%, n = 56), high screening cost (20.5%, n = 41), and fear of results (18.0%, n = 36). A total of 149 (74.5%) participants indicated willingness to undergo screening if it were free. Difficulty accessing screening services was reported by 49 (24.5%) participants, and 20 (10.0%) stated screening was not available in their area. Regarding stigma, 44 (22.0%) participants believed it negatively influenced screening uptake. Most respondents (54.5%, n = 109) viewed regular screening for high-risk individuals as very important. Suggested supports to improve screening included more awareness campaigns (43.0%, n = 86) and financial subsidies (31.5%, n = 63).

Conclusions

This study highlights significant barriers to lung cancer screening among high-risk populations in Saudi Arabia, including limited awareness, fear of diagnosis, cost, and access challenges. Interventions such as national awareness campaigns, cost reduction strategies, mobile screening units, and stigma mitigation are essential to improve screening uptake and reduce lung cancer mortality in the region.

## Introduction

Lung cancer is one of the leading causes of cancer-related morbidity and mortality worldwide, with smoking being the most significant risk factor [1]. Despite advances in treatment, the prognosis for lung cancer remains poor, largely due to the late-stage diagnosis at the time of symptom presentation. Early detection through screening has the potential to reduce lung cancer mortality, particularly among high-risk populations, such as smokers and individuals with a family history of lung cancer [2-4]. However, timely and widespread participation in lung cancer screening programs remains a challenge in many regions, including Saudi Arabia.

Lung cancer screening typically involves low-dose computed tomography (CT), a non-invasive imaging technique that has been shown to reduce lung cancer mortality by detecting cancers at an earlier, more treatable stage [4,5]. Several studies, including those conducted in high-income countries, have demonstrated the efficacy of low-dose CT screening in high-risk populations [3-6]. Despite the proven benefits, participation in lung cancer screening programs remains suboptimal due to a variety of barriers, both individual and systemic.

Barriers to lung cancer screening can be categorized into several domains, including knowledge and awareness, healthcare access, economic factors, and psychological concerns [2-7]. A lack of awareness about screening programs and the benefits of early detection is a major barrier in many populations. In addition, the cost of screening, both financial and logistical, can deter individuals from seeking screening, especially in low- to middle-income settings [4,8]. Psychological factors, such as fear of a cancer diagnosis, stigma associated with lung cancer (particularly among smokers), and a general sense of fatalism, can also reduce the likelihood of individuals participating in screening programs. Furthermore, healthcare access, including availability of screening facilities, geographic barriers, and the time commitment required for screening, also plays a critical role in determining whether individuals pursue timely screening [5,9].

This study aims to investigate the barriers to timely lung cancer screening in high-risk populations in Saudi Arabia. By examining the level of awareness, perceived barriers, healthcare access, and personal attitudes toward screening, the study seeks to identify the key factors that prevent individuals from participating in screening programs. The results of this study will provide valuable insights into how to improve lung cancer screening uptake, particularly in high-risk groups, and inform strategies for reducing the burden of lung cancer in Saudi Arabia.

## Materials and methods

Study design and participants

This study employed a cross-sectional survey design to assess barriers to timely lung cancer screening in high-risk populations. The survey was conducted among individuals residing in Saudi Arabia, with the primary focus on those at high risk of developing lung cancer. Inclusion criteria included individuals aged 18 years and older who had either a smoking history or a family history of lung cancer. Participants were excluded if they had previously been diagnosed with lung cancer, were currently undergoing treatment for any form of cancer, or had severe cognitive or language barriers preventing them from completing the survey. The participants were selected using convenience sampling from several healthcare centers across Saudi Arabia, ensuring a broad representation from both urban and rural areas.

Survey development

The survey was designed to collect data on demographic characteristics, knowledge and awareness of lung cancer screening, perceived barriers to screening, access to healthcare services, personal opinions, and suggestions for improving lung cancer screening in high-risk populations. The questionnaire consisted of 30 multiple-choice questions, with a mix of closed-ended questions and Likert scale items (Appendices). The survey was developed based on existing literature on lung cancer screening, consultations with oncologists, and expert opinion [2-4]. A pilot test was conducted with 20 participants to assess the clarity and relevance of the questions, leading to minor revisions before the final survey distribution.

The questionnaire was divided into the following six sections: demographic information (e.g., age, gender, occupation, smoking status, family history of lung cancer); knowledge and awareness of lung cancer screening (e.g., familiarity with screening programs, recommended age for screening); barriers to lung cancer screening (e.g., cost, access, fear of results, lack of awareness); healthcare access (e.g., availability of screening facilities, willingness to travel for screening); personal perception and opinions (e.g., stigma associated with screening, perceived importance of regular screening); and additional support for screening (e.g., support for awareness campaigns, financial support, mobile screening units).

Data collection

The data were collected between January and March 2025 through a combination of in-person interviews and self-administered surveys. Trained research assistants were stationed at the selected healthcare centers to distribute and assist with the completion of the surveys. Participants were informed about the objectives of the study and provided written consent before participating. Ethical approval for the study was obtained from the Ministry of Health, Saudi Arabia (approval number: 2024-12072), in accordance with the Declaration of Helsinki.

Variables and measures

Demographic variables included age, gender, occupation, smoking status, and family history of lung cancer. Knowledge and awareness of lung cancer screening were measured through questions regarding the participant’s familiarity with screening programs, the recommended age for screening, and the specific tests used for screening. Barriers to screening were assessed by asking participants about various obstacles, such as cost, access to screening facilities, and fear of results. Healthcare access was evaluated by asking about the availability of screening services, the ease of access, and the willingness to travel for screening. Personal perception and opinions included questions about perceived stigma related to lung cancer screening and the perceived importance of regular screening. Finally, additional support was measured by asking participants to identify what kind of support they believed would increase participation in lung cancer screening programs.

Sample size calculation

The sample size was calculated using a standard formula for cross-sectional surveys, considering an estimated awareness prevalence of 50% (to ensure maximum variability), a 95% confidence level, and a 7% margin of error. Based on these parameters, the minimum required sample size was calculated to be 196 participants. To account for potential incomplete or missing responses, we aimed to recruit at least 200 participants, which was achieved in the final sample.

Statistical analysis

Data were analyzed using SPSS version 26.0 (IBM Corporation, Armonk, NY, USA). Descriptive statistics were calculated for all variables, including frequencies, percentages, and means. Demographic characteristics were presented as counts and percentages. The data were checked for missing responses, and any incomplete surveys were excluded from the analysis. The internal consistency of the survey was assessed using Cronbach’s alpha, which was found to be 0.82, indicating good reliability.

## Results

Demographic characteristics of participants

A total of 200 participants were included in the study, with a gender distribution of 112 (56.0%) males and 88 (44.0%) females. The age distribution showed that 22 (11.0%) participants were aged 18-30 years, 34 (17.0%) were aged 31-40 years, 45 (22.5%) were aged 41-50 years, 53 (26.5%) were aged 51-60 years, 31 (15.5%) were aged 61-70 years, and 15 (7.5%) were aged 71 years and above. Regarding occupation, most participants were in skilled labor (31.0%), followed by office workers (26.0%), and healthcare professionals (19.0%). Regarding a family history of lung cancer, 48 (24.0%) participants reported having a family history, while the majority (144 participants, 72.0%) did not. Smoking status showed that 83 (41.5%) participants were current smokers, 59 (29.5%) were former smokers, and 58 (29.0%) had never smoked (Table 1).

**Table 1:** Participant demographics and smoking history (N = 200). Data are presented as the number of participants (n) and percentages (%).

Characteristic		n (%)
Age (years)	18–30	22 (11.0%)
	31–40	34 (17.0%)
	41–50	45 (22.5%)
	51–60	53 (26.5%)
	61–70	31 (15.5%)
	71 and above	15 (7.5%)
Gender	Male	112 (56.0%)
	Female	88 (44.0%)
Occupation	Healthcare professional	38 (19.0%)
	Office worker	52 (26.0%)
	Skilled labor	62 (31.0%)
	Unemployed	23 (11.5%)
	Retired	12 (6.0%)
	Student	10 (5.0%)
	Other	3 (1.5%)
Family history of lung cancer	Yes	48 (24.0%)
	No	144 (72.0%)
	Don’t know	8 (4.0%)
Smoking status	Current smoker	83 (41.5%)
	Former smoker	59 (29.5%)
	Never smoked	58 (29.0%)

Knowledge and awareness of lung cancer screening

In terms of awareness of lung cancer screening programs, 72 (36.0%) participants were fully aware, 65 (32.5%) were somewhat aware, and 63 (31.5%) were not aware at all. Regarding the recommended age for lung cancer screening, 115 (57.5%) participants knew the appropriate age, while 61 (30.5%) did not, and 24 (12.0%) were unsure. As for the specific tests used for lung cancer screening, 107 (53.5%) participants were aware of the tests, 74 (37.0%) were not aware, and 19 (9.5%) were unsure (Table 2).

**Table 2:** Awareness and knowledge of lung cancer screening (N = 200). Data are presented as the number of participants (n) and percentages (%).

Question		n (%)
Are you aware of lung cancer screening programs?	Fully aware	72 (36.0%)
	A little aware	65 (32.5%)
	Not aware	63 (31.5%)
Do you know the recommended age for lung cancer screening?	Yes	115 (57.5%)
	No	61 (30.5%)
	Not sure	24 (12.0%)
Do you know what tests are used for lung cancer screening?	Yes	107 (53.5%)
	No	74 (37.0%)
	Not sure	19 (9.5%)

Barriers to lung cancer screening

The primary barriers to lung cancer screening identified by participants included a lack of awareness about screening (28.0%), high cost of screening (20.5%), and fear of results (18.0%). Other barriers included lack of access to screening facilities (12.0%), the absence of symptoms (8.0%), and cultural or social stigma (5.5%). When asked whether they would undergo lung cancer screening if it were available at no cost, 149 (74.5%) participants said yes, 21 (10.5%) said no, and 30 (15.0%) were uncertain. Regarding the necessity of screening in asymptomatic individuals, 125 (62.5%) participants believed it was necessary, while 42 (21.0%) did not, and 33 (16.5%) were unsure. Motivating factors for undergoing screening included knowledge of the importance of early detection (41.0%), a doctor’s recommendation (24.5%), and free screening availability (23.5%) (Table 3).

**Table 3:** Reported barriers and motivators for lung cancer screening (N = 200). Data are presented as the number of participants (n) and percentages (%).

Question		n (%)
Main barrier to lung cancer screening in your community	Lack of awareness about screening	56 (28.0%)
	High cost of screening	41 (20.5%)
	Fear of results (e.g., being diagnosed with cancer)	36 (18.0%)
	Lack of access to screening facilities	24 (12.0%)
	Lack of symptoms, so people don’t see the need	16 (8.0%)
	Cultural or social stigma about cancer	11 (5.5%)
	Other	16 (8.0%)
Would you undergo lung cancer screening if it were available at no cost?	Yes	149 (74.5%)
	No	21 (10.5%)
	Maybe	30 (15.0%)
Do you believe lung cancer screening is necessary if you feel healthy and do not have symptoms?	Yes	125 (62.5%)
	No	42 (21.0%)
	Not sure	33 (16.5%)
What would motivate you to undergo lung cancer screening?	Knowledge of the importance of early detection	82 (41.0%)
	Doctor’s recommendation	49 (24.5%)
	Free screening availability	47 (23.5%)
	Hearing about someone else’s experience	14 (7.0%)
	Personal family history of cancer	7 (3.5%)
	Other	1 (0.5%)

Healthcare access

Access to lung cancer screening varied among participants. Of the respondents, 39 (19.5%) participants found it very easy to access screening, 92 (46.0%) found it somewhat easy, and 49 (24.5%) found it not easy. A further 20 (10.0%) participants indicated that screening was not available in their area. In terms of travel willingness, 87 (43.5%) participants were willing to travel within 10 kilometers for screening, 58 (29.0%) would travel 10-20 kilometers, and 40 (20.0%) would travel more than 20 kilometers. A majority of 122 (61.0%) participants expressed willingness to undergo screening even if it required taking time off work or other daily activities, while 41 (20.5%) said no, and 37 (18.5%) were unsure. When asked about healthcare affordability, 98 (49.0%) participants considered healthcare services in their area somewhat affordable, 58 (29.0%) found them not affordable, and 32 (16.0%) considered them very affordable (Table 4).

**Table 4:** Access to lung cancer screening and perceived affordability (N = 200). Data are presented as the number of participants (n) and percentages (%).

Question		n (%)
How easy is it for you to access lung cancer screening in your area?	Very easy	39 (19.5%)
	Somewhat easy	92 (46.0%)
	Not easy	49 (24.5%)
	Not available	20 (10.0%)
If you needed lung cancer screening, how far would you be willing to travel to get it?	Within 10 kilometers	87 (43.5%)
	10–20 kilometers	58 (29.0%)
	More than 20 kilometers	40 (20.0%)
	I would not travel for screening	15 (7.5%)
How would you rate the cost of healthcare services in your area?	Very affordable	32 (16.0%)
	Somewhat affordable	98 (49.0%)
	Not affordable	58 (29.0%)
	Don’t know	12 (6.0%)
Would you be willing to undergo lung cancer screening if it required taking time off work or other daily activities?	Yes	122 (61.0%)
	No	41 (20.5%)
	Maybe	37 (18.5%)

Personal perception and opinions

Regarding the stigma associated with lung cancer screening, 44 (22.0%) participants believed there was a stigma, while 115 (57.5%) did not perceive any stigma, and 41 (20.5%) were unsure. When asked about the importance of regular lung cancer screening in high-risk individuals, 109 (54.5%) participants considered it very important, 61 (30.5%) considered it somewhat important, and 30 (15.0%) did not consider it important (Table 5).

**Table 5:** Perceptions of lung cancer screening and associated stigma (N = 200). Data are presented as the number of participants (n) and percentages (%).

Question		n (%)
Do you believe there is a stigma attached to lung cancer screening?	Yes	44 (22.0%)
	No	115 (57.5%)
	Not sure	41 (20.5%)
How important do you think it is to screen for lung cancer regularly if you’re at high risk?	Very important	109 (54.5%)
	Somewhat important	61 (30.5%)
	Not important	30 (15.0%)

Additional support for screening

Participants identified several forms of additional support that would help them in obtaining lung cancer screening. The most commonly cited support was more awareness campaigns (43.0%), followed by financial support or subsidies (31.5%). Other forms of support included access to mobile screening units (11.5%), information from healthcare providers (8.0%), and other unspecified forms (6.0%) (Table 6).

**Table 6:** Preferred support mechanisms to improve screening uptake (N = 200). Data are presented as the number of participants (n) and percentages (%).

Question		n (%)
What additional support would help you in getting screened for lung cancer?	More awareness campaigns	86 (43.0%)
	Financial support or subsidies	63 (31.5%)
	Access to mobile screening units	23 (11.5%)
	Information from healthcare providers	16 (8.0%)
	Other	12 (6.0%)

## Discussion

This cross-sectional survey aimed to identify barriers to timely lung cancer screening among high-risk populations in Saudi Arabia. Our results highlighted several significant factors influencing individuals’ participation in lung cancer screening programs, particularly among those with a smoking history or a family history of lung cancer.

First, the study found that while the majority of participants were at least somewhat aware of lung cancer screening programs, a substantial proportion of the population remains unaware of both the availability and necessity of screening. Only 36.0% of participants were fully aware of lung cancer screening programs, and 12.0% were unaware of the recommended age for screening. This indicates a notable gap in public education and outreach, suggesting that more awareness campaigns are crucial in addressing these knowledge deficits.

The most significant barriers to screening identified by participants were a lack of awareness, high cost, and fear of the results. This is consistent with findings from other studies that show financial constraints and psychological factors, such as fear or anxiety about cancer diagnoses, often prevent individuals from seeking timely screening. The cost of screening was identified as a barrier by 20.5% of participants, which aligns with previous research indicating that financial burdens can restrict access to healthcare services in high-risk groups, especially in low- to middle-income countries. Additionally, a surprising finding was the prevalence of participants who would undergo screening if it were offered at no cost. A substantial 74.5% of participants expressed willingness to participate in lung cancer screening if cost were not a factor. This underscores the need for policy changes that would remove financial barriers to screening, such as implementing subsidized or free screening programs for high-risk individuals.

Our study confirmed that many participants perceived a lack of awareness and fear of results as critical barriers to screening. More than a quarter of participants cited lack of awareness as the primary obstacle, which is in line with prior studies that have highlighted the role of education in increasing screening uptake. Interestingly, fear of the results was identified by 18.0% of participants as a significant barrier, suggesting that emotional concerns may influence decision-making. Psychological factors related to cancer screening, including fear of a positive diagnosis, have been well-documented and could be addressed through counseling and emotional support services at the point of screening [10-13].

Moreover, the absence of symptoms was another barrier to screening, with 8.0% of participants indicating they did not feel the need to be screened because they were asymptomatic. This perception could potentially delay screening in individuals who might benefit from early detection. Several studies have emphasized the importance of educating individuals about the benefits of early detection, even in the absence of symptoms, particularly for high-risk populations.

When it comes to healthcare access, the study found that a majority of participants (61.0%) expressed willingness to undergo screening even if it required taking time off work or other daily activities. This is promising, as it suggests that, when given sufficient motivation, individuals may be willing to overcome logistical challenges to seek screening. However, access to screening facilities was still a significant concern, with 24.5% of participants reporting difficulty accessing screening services. While some respondents (19.5%) found it very easy to access screening, the fact that 10.0% of participants indicated that screening was not available in their area suggests a need for expanding screening programs to underserved regions. Mobile screening units and telemedicine could serve as valuable solutions to improve access for those living in remote areas or for those unable to travel long distances for screening [9-15].

In our study, 43.5% of participants were willing to travel within 10 kilometers for screening, and 29.0% were willing to travel up to 20 kilometers. This finding indicates a potential opportunity to target specific geographic areas where the availability of screening services is limited, offering outreach programs or mobile clinics in areas with high numbers of high-risk individuals.

A key issue identified in this study was the stigma associated with lung cancer screening, with 22.0% of participants believing that stigma prevented people from seeking screening. This is consistent with previous studies that have highlighted the social stigma surrounding lung cancer, particularly among smokers or individuals with a history of smoking. Participants’ attitudes toward lung cancer screening may be influenced by cultural beliefs and social norms that associate the disease with personal behavior (such as smoking). These findings suggest that future public health campaigns should not only focus on raising awareness about the medical benefits of screening but also work to address stigma and improve public attitudes toward the disease. This could include promoting lung cancer screening as a preventive health measure rather than focusing on the disease as a consequence of smoking or risky behaviors [14,16].

The majority of participants in this study considered lung cancer screening to be important, especially for those at high risk. Overall, 54.5% of participants rated the importance of regular screening as “very important,” reflecting growing recognition of the benefits of early detection. However, a significant proportion of respondents (30.5%) considered it somewhat important, and 15.0% did not consider it important at all. This variation suggests that further education is needed to emphasize the life-saving potential of early detection in high-risk populations. In particular, the importance of screening should be highlighted in healthcare settings where high-risk individuals regularly seek care, such as smoking cessation clinics or oncology clinics.

Based on our findings, several recommendations can be made to improve lung cancer screening rates among high-risk populations in Saudi Arabia. First, national health policies should prioritize the implementation of low-cost or free screening programs, especially for individuals at high risk, such as smokers and those with a family history of lung cancer. Additionally, public health initiatives should aim to increase awareness of the importance of early screening, specifically targeting high-risk populations through tailored educational campaigns. These campaigns should not only focus on the medical benefits of screening but also address emotional and psychological barriers, such as fear and stigma, by offering counseling and support services. Furthermore, the accessibility of screening programs must be expanded, particularly in rural areas, by deploying mobile screening units or offering telemedicine-based services for consultations and follow-ups. Lastly, efforts should be made to reduce the stigma associated with lung cancer, as this could further encourage individuals to participate in screening programs without the fear of judgment.

Limitations

This study has several limitations. First, the convenience sampling method used to recruit participants may limit the generalizability of the findings. The sample may not fully represent the broader population of high-risk individuals across Saudi Arabia. Additionally, self-reported data are prone to response biases, including social desirability bias and recall bias, which could have influenced the accuracy of the responses. Finally, while the survey provided valuable insights into the barriers to lung cancer screening, it did not assess clinical factors, such as the participants’ actual risk level or medical history, which could have provided more detailed context for their attitudes and behaviors toward screening.

## Conclusions

This study highlights the significant barriers to timely lung cancer screening in high-risk populations in Saudi Arabia, including a lack of awareness, fear of results, and financial constraints. These findings underscore the urgent need for targeted interventions aimed at increasing public awareness, reducing financial and logistical barriers, and addressing psychological concerns such as fear and stigma. By improving access to screening services and promoting education on the importance of early detection, particularly among high-risk individuals, it is possible to enhance participation in lung cancer screening programs. Such efforts could ultimately contribute to earlier diagnosis, improved treatment outcomes, and a reduction in lung cancer mortality rates in Saudi Arabia. Future research should focus on evaluating the effectiveness of these interventions and expanding screening programs to ensure that all individuals, particularly those in underserved areas, can benefit from timely lung cancer detection.

## Appendices

Survey questionnaire

**Table 7:** Lung cancer screening questionnaire for high-risk individuals.

Section	Question	Answer choices
Demographic information	What is your age?	18–30 / 31–40 / 41–50 / 51–60 / 61–70 / 71 and above
	What is your gender?	Male / Female
	What is your current occupation?	Healthcare professional / Office worker / Skilled labor / Unemployed / Retired / Student / Other (specify)
	Do you have a family history of lung cancer?	Yes / No / Don’t know
	What is your smoking status?	Current smoker / Former smoker / Never smoked
Knowledge and awareness	Are you aware of lung cancer screening programs?	Fully aware / A little aware / Not aware
	Do you know the recommended age to start lung cancer screening?	Yes / No / Not sure
	Do you know what tests are used for lung cancer screening?	Yes / No / Not sure
Barriers to lung cancer screening	What is the main barrier to lung cancer screening in your community?	Lack of awareness / High cost / Fear of results / Lack of access / Lack of symptoms / Cultural or social stigma / Other (specify)
	Would you undergo lung cancer screening if it were available at no cost?	Yes / No / Maybe
	Do you believe lung cancer screening is necessary if you feel healthy and have no symptoms?	Yes / No / Not sure
	What would motivate you to undergo lung cancer screening?	Knowledge of early detection / Doctor’s recommendation / Free screening availability / Hearing about someone’s experience / Family history / Other (specify)
Healthcare access	How easy is it for you to access lung cancer screening in your area?	Very easy / Somewhat easy / Not easy / Not available
	If you needed lung cancer screening, how far would you be willing to travel?	Within 10 km / 10–20 km / More than 20 km / Would not travel
	How would you rate the cost of healthcare services in your area?	Very affordable / Somewhat affordable / Not affordable / Don’t know
	Would you be willing to undergo lung cancer screening if it required taking time off work or daily activities?	Yes / No / Maybe
Personal perception and opinions	Do you believe there is a stigma attached to lung cancer screening?	Yes / No / Not sure
	How important do you think regular screening is if you are high-risk?	Very important / Somewhat important / Not important
Additional support for screening	What additional support would help you in getting screened?	More awareness campaigns / Financial support or subsidies / Mobile screening units / Information from healthcare providers / Other (specify)
